# Giant aneurysm of the right intra thoracic sub-clavian artery presenting as a dysphonia

**DOI:** 10.4314/pamj.v9i1.71217

**Published:** 2011-08-17

**Authors:** Alae Mechchat, Massine M El Hammoumi, Abbas EL Mesnaoui, Brahim Lekehal, Youness Bensaid

**Affiliations:** 1 Department D of General and Vascular Surgery, Hospital Avicenne, Rabat, Morocco; 2 Department of Thoracic Surgery, Military University Hospital, Rabat, Morocco

**Keywords:** Aneurysm, sub-clavian artery, surgery, Morocco

## Abstract

Aneurysms of the intra-thoracic subclavian artery (SCA) are rare. They are often revealed by complications. Surgical treatment is always indicated. Endovascular treatment is a less invasive alternative. We report a case of a 60 years-old woman admitted for right chest pain and dysphonia. Laryngoscopy noted a right vocal cord palsy. Chest computed tomography and angiography showed a giant aneurysm of the intra-thoracic right SCA. A resection-ligation of the aneurysm was performed by a supra-clavicular approach. Postoperative course was uneventful. The histology defined an atherosclerotic aneurysm. The patient underwent voice reeducation with partial improvement after six months.

## Introduction

Arterial Aneurysms of the intra-thoracic segment of sub-clavian artery are rare and often asymptomatic. Very few cases have been reported in the literature. The prognosis is still dominated by the risk of thrombo-embolic complications and lethal rupture. We report a case of a 65 years old female with an atherosclerotic aneurysm of right intra-thoracic sub-clavian artery presenting as a palsy of the recurrent laryngeal nerve.

## Case report

We report the case of a 60 years old female, with past medical history of hypertension, without previous chest trauma or pulmonary infection. She had a six months history of dyspnea and chest pain associated with a progressive dysphonia.

Clinical examination on admission objectified a stable hemodynamic status. Neurological examination was unremarkable. The vascular examination noted a weak right radial pulse compared to the left side without signs of ischemia of the right hand. No pulsatile supra- clavicular mass was found.

**Figure 1:**
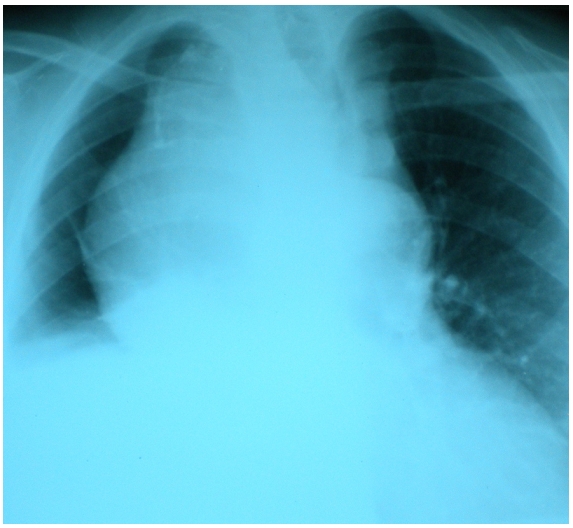
Chest radiograph showing a giant right mediastinal mass with tracheal shift

Chest examination showed a right basal condensation. Laryngoscopy confirmed a right vocal cord palsy that explained the dysphonia. Chest radiography showed a giant mass of the right hemithorax, with mediastinal shift and right basal lung atelectasis (Figure 1).

**Figure 2:**
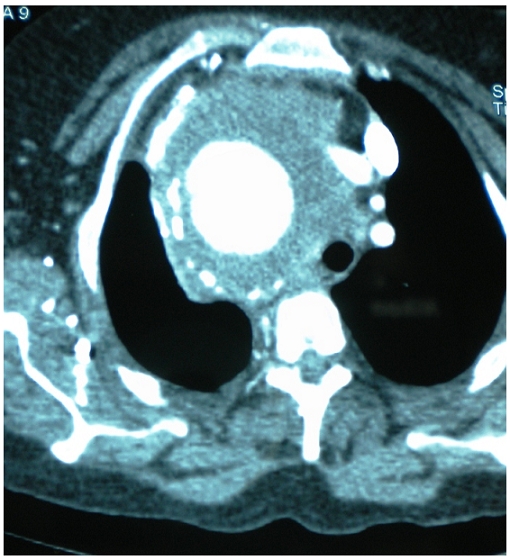
Angio-computed-tomography: an intra-thoracic right sub-clavian artery aneurysm with supra aortic vessels shift

**Figure 3:**
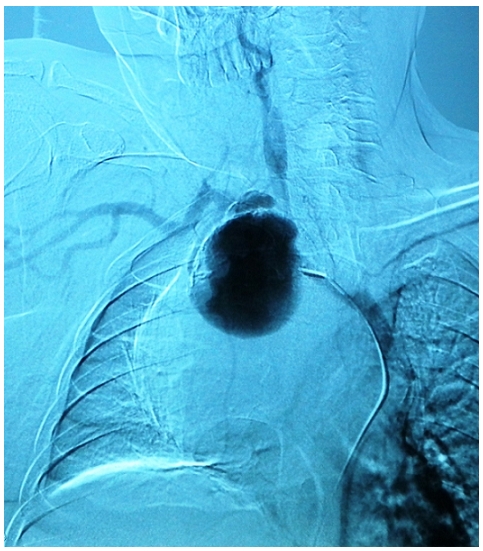
Angiography-a saccular aneurysm of the intra thoracic segment of the right sub-clavian artery with wall thrombosis

**Figure 4:**
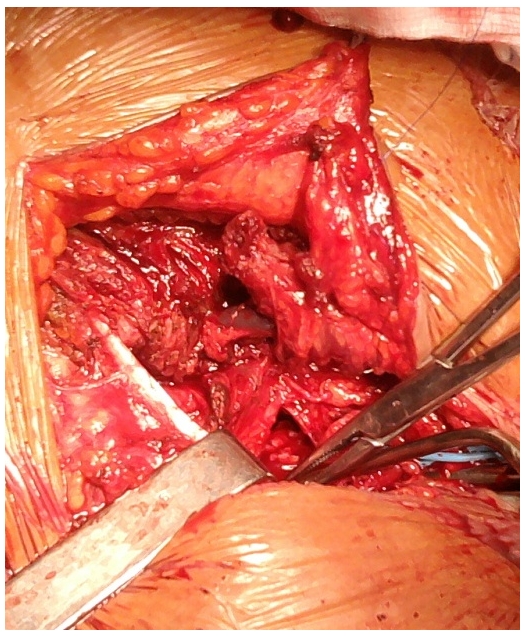
Per operative view of the aneurysm after ligature of the proximal segment of the sub clavian artery (SCA: sub clavian artery, SCV:subclavian vein, SCM: sternocleidomastoideus)

The Doppler ultrasounds, angiography of the supra aortic vessels and thoracic Computed Tomography concluded to a saccular aneurysm of the right intra-thoracic sub-clavian artery (RSA) with partial thrombosis. The aneurysm measured 10 cm in diameter (Figure 2). The CT noted also a right lower lobe atelectasis. The angiography defined the aneurysm beginning a few centimeters after the origin of the right sub-clavian artery (Figure 3). The ECG and echocardiogram revealed hypertensive heart disease. The patient underwent a supraclavicular approach and right claviculo-sternal dislocation. After laborious dissection of the aneurysm due to severe adhesions we debrided thrombotic material. Per operatively we were forced to leave undetached wall aneurysm and we couldn't find the recurrent laryngeal nerve (Figure 4). The pulse of the distal segment of the sub-clavian artery was satisfactory so we performed a simple ligature of aneurysm loops without end-to-end anastomosis. The postoperative course was uneventful. Cultures from thrombus and the wall of the aneurysm were negative. Pathology confirmed the atherosclerotic origin of the aneurysm. The patient was admitted for voice reeducation. After six month of follow-up the patient showed a partial improvement.

## Discussion

Aneurysms of the subclavian artery is about 1% of peripheral aneurysms [1, 2]. They can be classified into two groups according to their different etiologies, presentations and treatment: the intrathoracic and extrathoracic subclavian artery aneurysms. While extrathoracic aneurysms are often related to a thoracic outlet syndrome or an old trauma, intra-thoracic aneurysms are often caused by atherosclerotic disease [3] as in our reported case, more rarely fibrodysplastic, infectious or traumatic etiologies.

Extrathoracic subclavian artery aneurysms presented classically as a supraclavicular pulsatile mass, those of the intrathoracic segment are often asymptomatic. They often presented at the stage of complications such as compression of adjacent structures: dysphagia if esophageal compression [4], dysphonia when recurrent laryngeal nerve compression [5] or underlying lung atelectasis. More rarely, the clinical presentation can be a thromboembolic complication such as ischemia of the upper limb. Finally, some aneurysms can be diagnosed after a rupture causing fatal hemorrhage.

At present, the standard management of subclavian artery aneurysms is surgical repair. Surgical attitude is always indicated even for asymptomatic aneurysms because of the risk of complications. A sternotomy is often necessary for aneurysms on the right side, while a lateral thoracotomy may be sufficient for aneurysms on the left side. Most of authors prefer resection to simple ligature [3, 6, 7]. In our case, the choice of the ligature was motivated by the suspicion of potentially infected intra-aneurysmal thrombus and persistent pulse in the distal subclavian artery. Currently, endovascular treatment by stent when it is anatomically possible, remains an attractive alternative since it allows a less invasive approach [8].

## Conclusion

Aneurysms of the intra-thoracic sub-clavian artery are unusual. They often present at the stage of complication such thrombo-embolic accidents, fatal rupture or compression of adjacent structures such as our reported case. The standard management is surgical. The endovascular approach can be a good alternative.
